# The Effect of *Ganoderma Microsporum* immunomodulatory proteins on alleviating PM_2.5_-induced inflammatory responses in pregnant rats and fine particulate matter-induced neurological damage in the offsprings

**DOI:** 10.1038/s41598-019-38810-5

**Published:** 2019-05-02

**Authors:** Chia-Yi Tseng, Jia-Yu Yu, Yu-Chen Chuang, Chia-Ying Lin, Chun-Hao Wu, Chia-Wei Liao, Fu-Hua Yang, Ming-Wei Chao

**Affiliations:** 1 Department of Biomedical Engineering, Chung Yuan Christian University, Zhongli district, Taoyuan, Taiwan 320; Chung Yuan Christian University, Zhongli district, Taoyuan, 32023 Taiwan; 2 Department of Bioscience Technology, Chung Yuan Christian University, Zhongli district, Taoyuan, Taiwan 320; Chung Yuan Christian University, Zhongli district, Taoyuan, 32023 Taiwan; 3 Center for Nanotechnology, Chung Yuan Christian University, Zhongli district, Taoyuan, Taiwan 320; Chung Yuan Christian University, Zhongli district, Taoyuan, 32023 Taiwan

**Keywords:** Development of the nervous system, Neurodevelopmental disorders

## Abstract

Fine particulate matter 2.5 (PM_2.5_) induces free radicals and oxidative stress in animals, leading to a range of illnesses. In this study, *Ganoderma Microsporum* immunomodulatory (GMI) proteins were administered to alleviate PM_2.5_-induced inflammatory responses in mother rats, and PM_2.5_-induced inflammatory responses and neurological damage in their offspring. The results suggested that GMI administration decreased the risk of neurological disorders in mother rats and their offspring by reducing the white blood cell count, lessening inflammatory responses and PM_2.5_-induced memory impairment, and preventing dendritic branches in the hippocampi from declining and microRNAs from PM_2.5_-induced modulation.

## Introduction

Rapid advances in technology have produced environmental pollution. In particular, fine particulate matter 2.5 (PM_2.5_) from industrial activities and vehicles has received increasing attention from the public. PM_2.5_ has been demonstrated to induce radicals and oxidative stress in animals, impairing dopamine neurons, RNAs, and DNAs, leading to neuritis, stroke, Alzheimer’s disease, and Parkinson’s disease^1^, and increasing purine and pyrimidine sites in the hippocampus to cause oxidative genetic damage^2,3^. Epidemiologists have revealed that PM_2.5_ impairs working memory^4,5^.

Moreover, PM_2.5_ can intensify oxidative stress in the blood of fetuses, precluding their growth^6^. Particulate matter also alters E18 fetal cytokine and modulates microRNA (miRNA) expression in the cortex and hippocampus^7^.

*Ganoderma lucidum*, a commonly used herb in traditional Chinese medicine, contains physiologically active, antioxidative, and immunological ingredients such as polysaccharides, peptidoglycans, and triterpenes^8^. In the present study, immunomodulatory proteins extracted from *G. Microsporum* (GMI) were used. Recent studies have suggested that GMI can inhibit reactive oxygen species in A549 cells and the invasion and transfer of tumor necrosis factor-alpha (TNF-α)^9^. Additionally, it can be combined with cisplatin as an adjuvant for cancer treatment^10^.

On the basis of this rationale, this study explored whether GMI alleviated the PM_2.5_ exposure-related harm in pregnant rats. In addition, PM_2.5_-induced inflammatory responses were examined, miRNA expression (which plays a role in fetal neurological disorders) was estimated, and a behavioral test was used to assess the memory capacity of offspring.

## Materials and Methods

### Chemical

The immunomodulatory protein is derived from *Ganoderma microsporum* (GMI), which is manufactured by Mycomagic Biotechnology Co., Ltd., (Taipei, Taiwan). The detail methods of GMI usage have been described previously^11^.

### Animals

The Institutional Animal Care and Use Committee of Chung Yuan Christian University approved the animal experiment protocol with number: 106012. We confirm that all experiments were performed in accordance with relevant guidelines and regulations. The four to six week-old Sprague-Dawley rats were obtained from BioLasco Taiwan Co., Ltd. Rats were obtained excellent care with standard 12/12 light/dark cycle with temperature at 18 to 26 °C and humidity at 30% to 70%. The rats were provided with fresh food and water. The cages and premises were kept very clean. After a week of acclimation, all rats were exposed in two different PM_2.5_ concentration (0 and 2.5 mg/m^3^) give instillation intratracheal test every other day. The instillation intratracheal protocols were according to the previous study design^12^. And GMI (0, 0.33, and 3.3 μg/kg daily) was delivered orally via a gavage needle in pregnancy until the E21.

### PM_2.5_ preparation

Particle (SKU-Pack Size: CRM558, Diesel - Clay Loam 1) were obtained from Sigma, USA. Stock suspensions (20 mg/mL) of particles were prepared with the protocol described previously^13^. The particles size was confirmed as PM_2.5_ with using dynamic light scatter.

### Morris water maze test

The Morris water maze (MWM) was a black circle pool (160 cm in diameter, 45 cm in deep). The pool was divided into four equal space quadrants. The pool was filled to a depth of 27 cm with water (25 ± 2 °C). A circle platform (12 cm in diameter, 25 cm in deep) was submerged 2 cm below water surface and placed at a fixed position in quadrant III, 50.8 cm from the wall. The camera was mounted above of the center of the pool and analyzed by Ethovision XT v 10.0 software. The acquisition trial was carried out during 4 consecutive days. The rats received 4 consecutive training trials on each training days. A different starting location was used for each trial, which consisted of swimming following by remaining on the platform for at least 20 sec. If rats could not reach to the platform within 60 sec, it was guided it to platform. Time to reach the platform (latency to sec) and the path length were measured. After acquisition trial, the platform was removed from the pool. The rats were allowed to swim 60 sec. First time to platform (latency time) were measured.

### Working memory test

Working memory test used as same as MWM pool. The platform changed every day during 6 experiment days. Once the rats training finished, that remaining on the platform for at least 20 sec. If rats could not reach to the platform within 60 sec, it was guided it to platform. Than allowed to swim 60 sec. After training, rats were allowed to swim 60 sec. First time to platform (latency time) were measured.

### Novel object and Location recognition test

Let rats in transparent acrylic box (100 cm each side, 50 cm height). In first two days allowed rats explore 5 min for training. Third days changed one object or changed object location, then allowed rats explore 5 min. Measured exploration ratio = explored novel object time/total explored two objects time.

### Brain immunostaining

Rats were perused with PBS to removed blood. The brain tissue soaked in FD Rapid Golgi Stain TM kit A and B miscible liquids at 1:1 and kept away from light in room temperature for 3 weeks. Then soaked in C solution in 4 °C for 48 hr. Then, the brains were embedded in OCT (optimal cutting temperatuew compound) and soaked in liquid nitrogen. Frozen sections were prepared at 80 μm thickness and fixed by C solution. The sections were soaked in D, E, and ddH_2_O miscible liquids at 1:1:2. Then, soaked in 50%, 75%, 95%, 100% EtOH, and Xylene. (Stain protocol were followed FD Rapid Golgi Stain TM kit manual).

### Bonfire neuron analysis

Bonfire is dendritic morphology analysis software that constructed by MATLAB^14,15^. Bonfire program has to apply with Neuron J plugin to ImageJ and Neuro Studio. First, using Image J to outline and record the neuron morphology, and then define the connection between axons and dendrites through NeuroStudio. After manually removing the tracing of axons, then make a specific analysis on dendritic morphology. The results can be converted by Bonfire program into other form, that can remove the wrong morphology record by semi-automatic tracking. And got Sholl and terminal/branching points results. Those resuls could convert into Excel for further analysis.

### White blood cell analysis

Analysis was performed using a rapid hematocrit solution (Askbiotech, Taiwan). The reagents were fixed solution (methanol and stabilizer), a staining solution (eosin), a staining solution II (methylene blue and azure dye). Eosint could let intracellular eosinophilic to be red, blue andazure dye could let intracellular basophilic part became blue. The staining results showed red blood cells were red; neutrophils nuclei were blue, cytoplasm were pink; eosinophilics nuclei were blue, the cytoplasm were blue, the particles were red; basophils nuclei were blue, dark purple particles; Monocytes nuclei were blue-purple, cytoplasm pale blue; lymphocytes nuclei were blue-purple and large and round, the cytoplasm was blue.

### Measurement of oxidative stress cytokines level in serum

Serum oxidative stress cytokines were detected by Rat oxidative stress kit (Signosis EA-1501). First, add 100 μl standard and ten-fold diluted serum to each well for 2 hr at room temperature, then wash three times. Added Biotin labeled antibody 100 μl for 1 hr at room temperature, then wash three times. And added Streptavidin HRP 100 μl at room temperature for 45 minutes. Finally read at 450 nm. (Process were followed Signosis Rat Oxidative Stress ELISA kit manual).

### Measurement of IgE level in Serum

IgE were detected by Anti-Ovalbumin IgE ELISA kit (Cayman, USA). First, add 100 μl standard and serum to each well for 2 hr at room temperature, then wash four times. Added ova-biotion conjugate 100 μl for 1 hr at room temperature, then wash four times. And added Streptavidin HRP 100 μl at room temperature for 30 minutes, then wash four times. Next add TMB for 30 min and HRP stop solution. Finally read at 450 nm. (Process were followed Cayman Anti-Ovalbumin IgE ELISA kit manual).

### Measurement of Histamine level in Serum

Histamine was detected by Histamine ELISA kit (Enzo, USA). First, add 100 μl standard and serum to each well for 1 hr at room temperature, and then wash three times. Added SA-HRP 200 μl for 30 mine at room temperature, then wash three times. Added TMB for 30 min and stop solution. Finally read at 450 nm. (Process were followed Enzo Histamine ELISA kit manual).

### Western Blot

Rats were euthanized via CO_2_ asphyxiation, and the brains were removed. Tissues were homogenized in TEE buffer with PMSF, and used Triton X-100 to lysis cell membrane. Lysates were centrifuged (13000 rpm at 4 °C) for 10 min, and the supernatants were collected. Protein concentrations were determined by BCA assay kit. Proteins were loaded 50 μg/well on a 12% SDS-polyacrylamide gel and transferred to a PVDF membrane. Nonspecific reativity was blocked for 1 h at room temperature and primary anti-CD68, anti-catalase, anti-SOD-1 and anti-GAPDH were incubated overnight at 4 °C. Then, the membranes were washed for 20 min for three times. Secondary antibodies conjugated with horseradish peroxidase (HRP) were used at 1:10000 dilution. Immunoreactive bands were detected using ECL system. The total intensity of bands were analyzed by ImageJ.

### miRNA analysis

Removed fetals brain tissue in E18, and divided into hippocampus and cortex then put into TRIzol.Then, used Rat Neurological Development & Disease miRNA PCR Array (Qiagen MIRN-107Z, Germany) to analysis neuron development and neuron disease. First, miRNA reverse- transcription. Second, analyzed miRNA by qPCR array.

### Statistics

All data was expressed as the mean ± SEM. Statistical significance (p < 0.05) between groups was determined using ANOVA following by the appropriate post hoc test or between two selected groups using appropriate test by GraphPad InStat software. *p < 0.05; **p < 0.01; ***p < 0.001.

## Results

### GMI alleviated the inflammatory responses in mother rats and their offspring for mother rats exposed to PM_2.5_ during pregnancy

**Figure 1:**
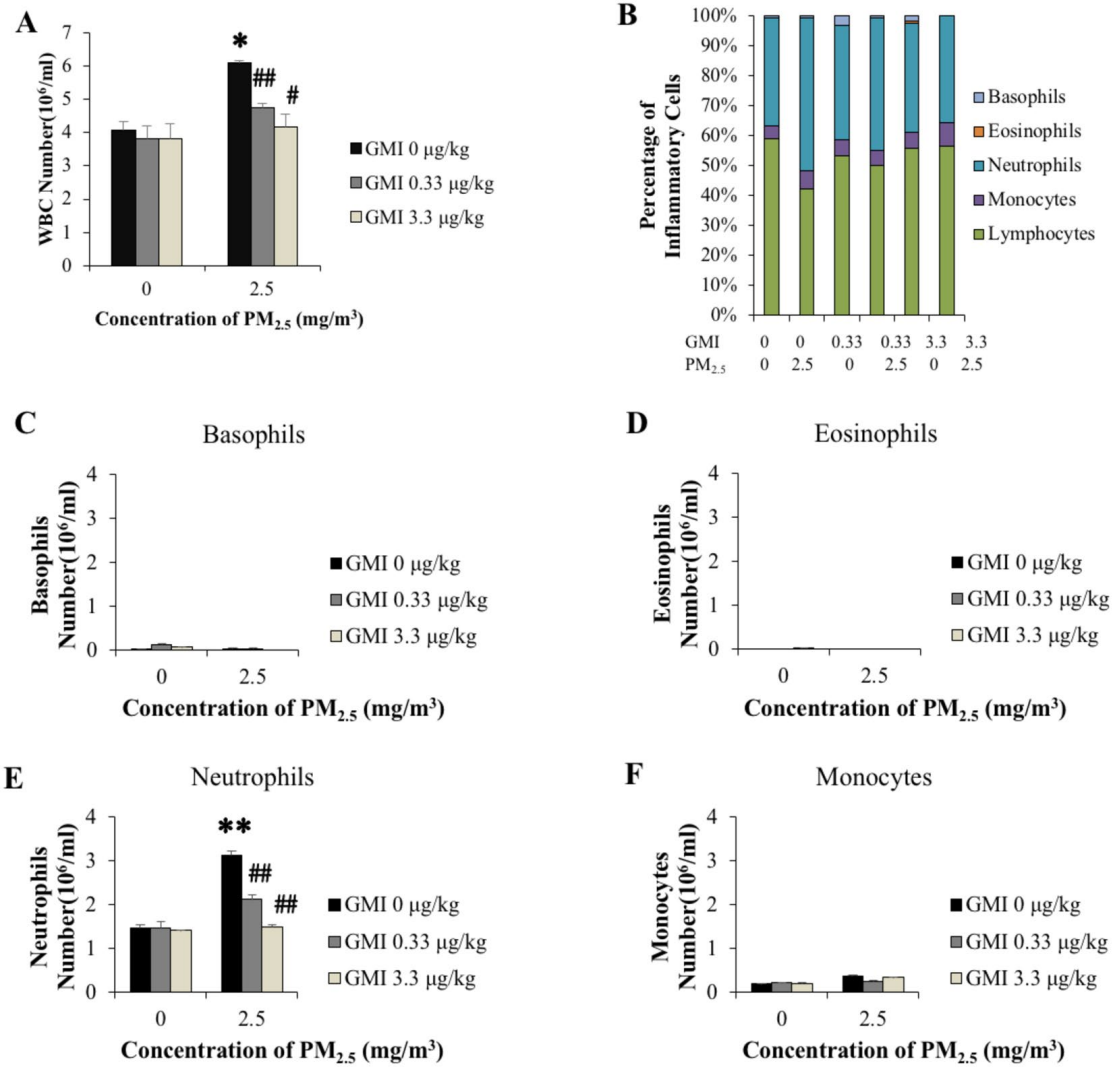
Immune cells raised after PM_2.5_ exposure during pregnancy. (**A**) PM_2.5_ increased Leukocyte number, addition of GMI decreased the level. (**B**) The percentage of immune cells. (**D**–**F**) Number of different inflammatory cells increased in response to PM_2.5_ exposure. GMI could decrease the inflammation of PM_2.5_. *, and ** indicate p < 0.05, and p < 0.01 significant difference, respectively, compared with control. ^#^, and ^##^ show p < 0.05, and p < 0.01, respectively, compared with PM_2.5_-treated controls.

**Figure 2:**
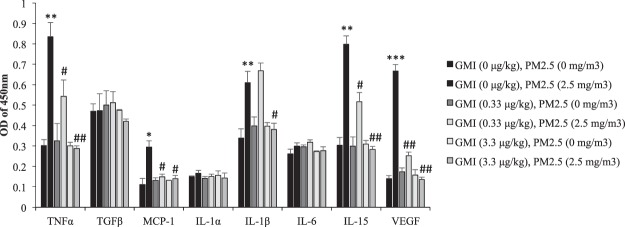
GMI decreased oxidative stress induced cytokines. Oxidative stress cytokines in serum was detected by Rat oxidative stress kit, which shows that GMI is able to mitigate the oxidative stress induced by PM_2.5_ exposure. *, and ** indicate p < 0.05, and p < 0.01 significant difference, respectively, compared with control. ^#^, and ^##^ show p < 0.05, and p < 0.01, respectively, compared with PM_2.5_-treated controls.

**Figure 3:**
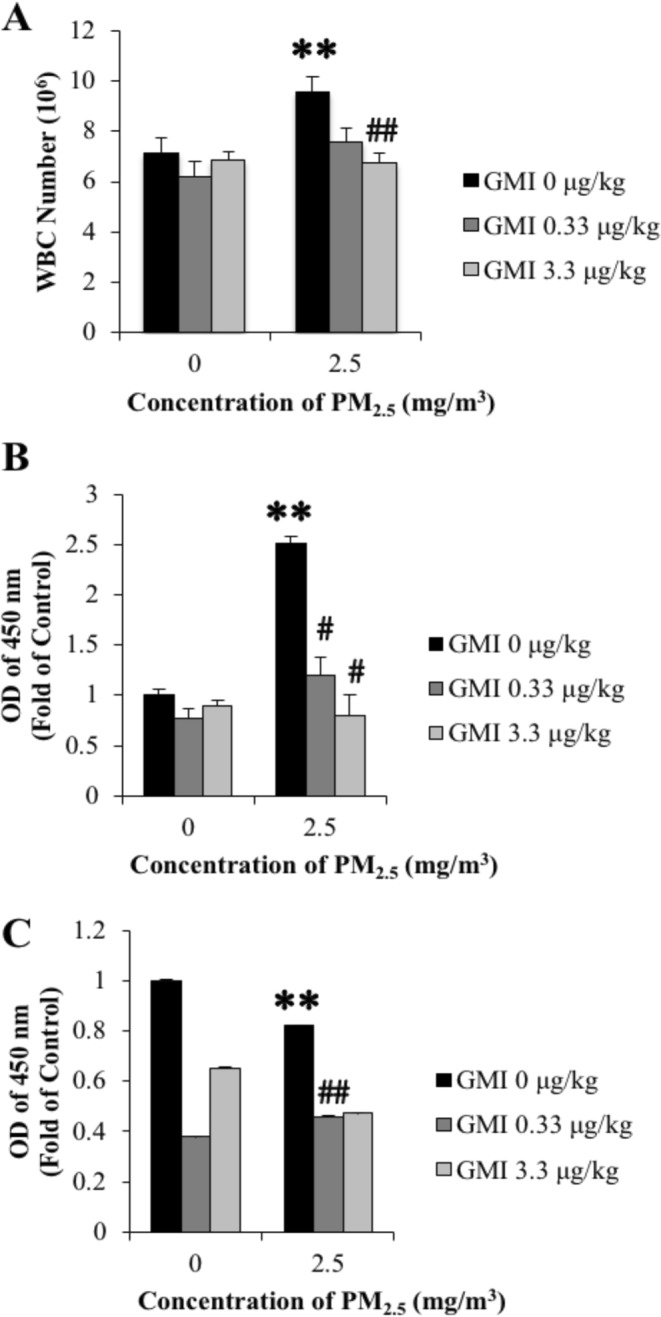
GMI reduce the PM_2.5_-caused inflammation in offsprings. (**A**) Leukocyte increased in offspring which maternal exposed to PM_2.5_, GMI decreased the level. (**B**) IgE (**C**) histamine level were restored in serum. *, and ** indicate p < 0.05, and p < 0.01 significant difference, respectively, compared with control. ^#^, and ^##^ show p < 0.05, and p < 0.01, respectively, compared with PM_2.5_-treated controls.

The white blood cell (WBC) count in pregnant rats after exposure to PM_2.5_, especially increased neutrophils; however, all types of WBCs decreased after GMI was administered in rats at different concentrations (Fig. 1). Exposure to PM_2.5_ also caused the expression of cytokines i.e., TNF-α, monocyte chemoattractant protein-1 (MCP-1), interleukin 1 beta (IL-1β), interleukin-15 (IL-15), and vascular endothelial growth factor (VEGF) significantly increased in the rats; however, GMI administration reduced the expression of these cytokines to levels comparable to control group rats (Fig. 2). These results indicated that GMI can alleviate inflammatory responses induced by exposure to PM_2.5_. Additionally, the WBC count, as well as immunoglobulin E (IgE) and histamine levels increased significantly in the offspring of female rats exposed to PM_2.5_ during pregnancy, although the offspring’s WBC count declined when their mothers were administered GMI during pregnancy (Fig. 3).

### GMI alleviated PM_2.5_-induced impairment of the memory capacity of offspring

**Figure 4:**
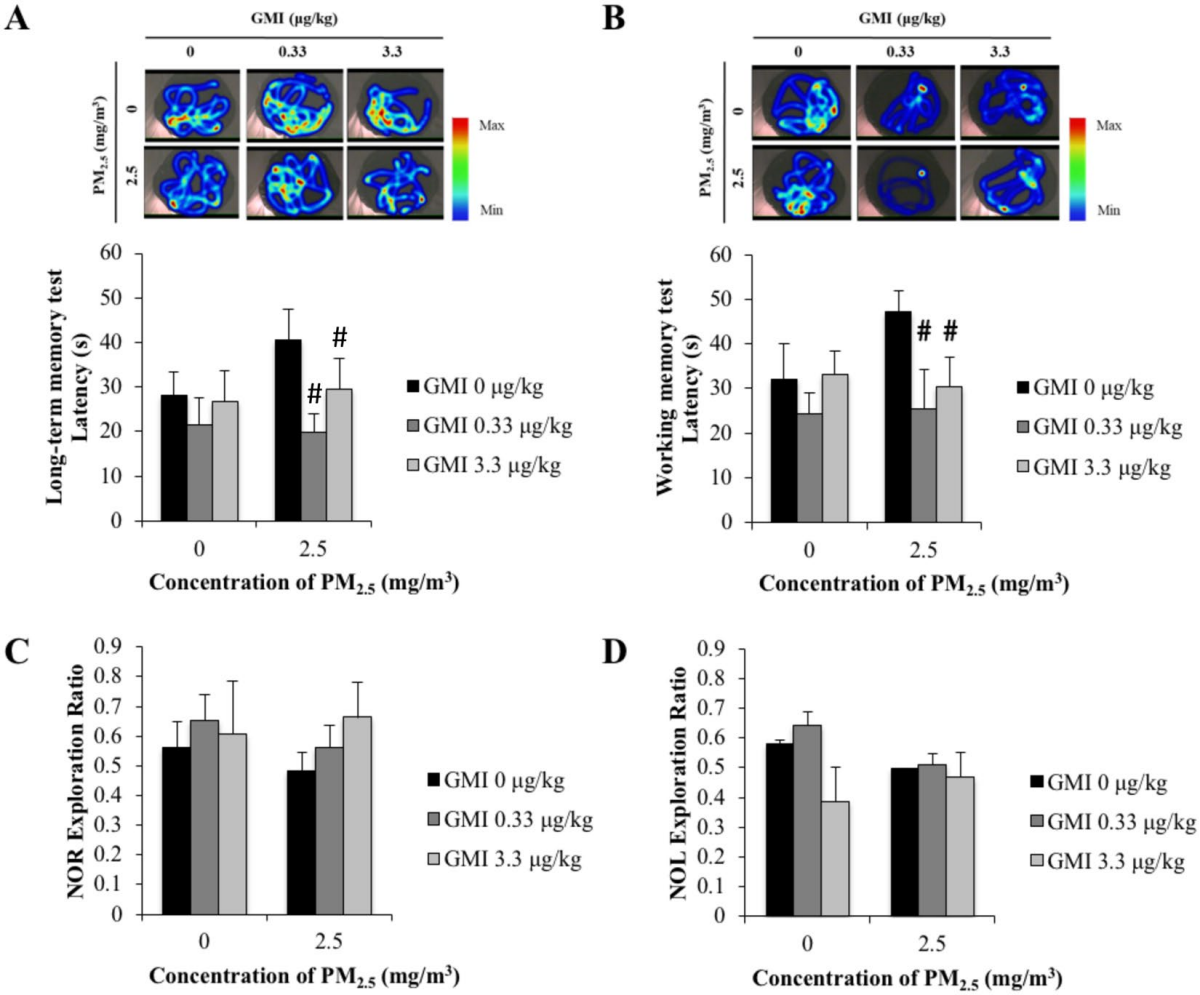
GMI treatment increase offspring’s working memory. MWM and NOL/NOR test of offspring. (**A**,**B**) PM_2.5_-exposed offspring swimming trail will not center on platform. GMI shorten the latency to platform in working memory test. (**C**,**D**) in NOR and NOL test PM_2.5_-exposed offspring spend less time to explore novel object. *, and ** indicate p < 0.05, and p < 0.01 significant difference, respectively, compared with control. ^#^, and ^##^ show p < 0.05, and p < 0.01, respectively, compared with PM_2.5_-treated controls.

The results of a behavioral tests indicated that the offspring of rats exposed to PM_2.5_ exhibited a decreased performance in recognition as well as long-term, working, and spatial memory (Fig. 4). However, PM_2.5_-induced damage to the memory capacity of the offspring of mother rats administered GMI during pregnancy was reduced, leading to significant differences in working memory in comparison with rat pups whose PM_2.5_-exposed mothers were not given GMI during pregnancy.

### GMI alleviated PM_2.5_-induced neurological impairments in offspring

**Figure 5:**
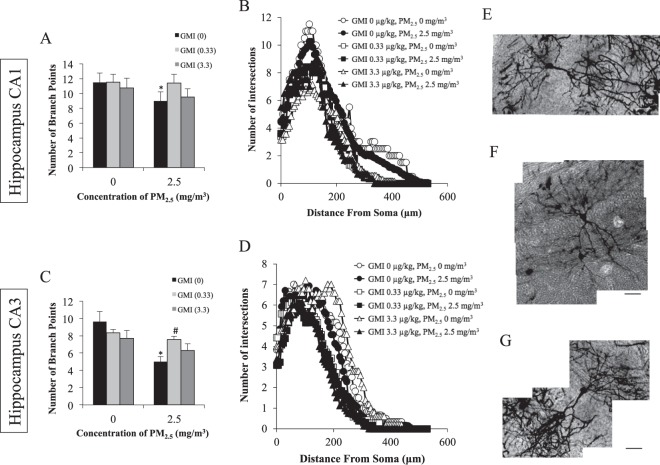
Maternal GMI treatment protect the neuron from PM_2.5_-caused neuronal damage in offspring hippocampus. Analysis of neuron branch points and distance of hippocampus by Bonfire program. (**A**,**C**) GMI increase the branch points in PM2.5 treated groups in CA3, and (**B**,**D**) the distance from dendrite intersections to neuronal soma was restored with GMI treatment. The representative images of neuron in CA1. (**E**) PM_2.5_ (0 mg/m^3^), GMI (0 μg/kg), (**F**) PM_2.5_ (2.5 mg/m^3^), GMI (0 μg/kg), (**G**) PM_2.5_ (2.5 mg/m3), GMI (0.33 μg/kg). *, and ** indicate p < 0.05, and p < 0.01 significant difference, respectively, compared with control. ^#^, and ^##^ show p < 0.05, and p < 0.01, respectively, compared with PM_2.5_-treated controls. The scale bar is 50 μg.

Cerebral neural cells were studied using the Golgi method, a heavy metal-staining procedure that examines the complexity of dendritic branches to determine the robustness of synaptic cells, and the capability of the cells to process and integrate messages. The quantitative results of the Sholl analysis (Fig. 5) revealed decreases in the number of dendritic branches and terminals in CA1 and CA3 of the hippocampus as a result of exposure to PM_2.5_. Therefore, exposure to PM_2.5_ decreased the number of dendritic branches and terminals. However, GMI attenuated this PM_2.5_-induced neurological damage.

### GMI caused microglia to decrease in the brains of offspring

**Figure 6:**
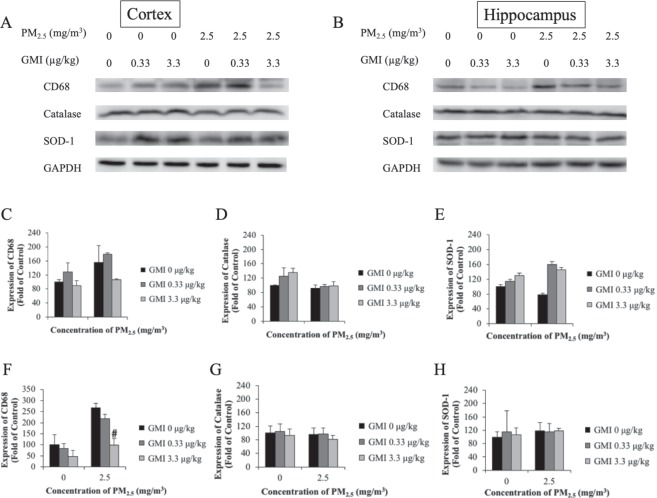
GMI decreased microglia activation which caused by maternal PM_2.5_ exposure. Immunoblot analysis determined the expression levels of microglia activation, catalase and SOD-1 in offspring cortex and hippocampus. PM_2.5_ increased microglia activation and GMI significantly reduced the microglia activation in hippocampus. *, and ** indicate p < 0.05, and p < 0.01 significant difference, respectively, compared with control. ^#^, and ^##^ show p < 0.05, and p < 0.01, respectively, compared with PM_2.5_-treated control.

The Western blot was performed on the cortex and hippocampus of offspring to estimate the expression of antioxidant proteins (SOD-1 and catalase) and macrophages/microglia (CD 68). With exposure to PM_2.5_, the expression of SOD-1 exhibited no significantly changed, whereas that CD68 increased, particularly in the cortex (Fig. 6). Moreover, after GMI administration, microglia activation rate decreased.

### GMI protected miRNAs from PM_2.5_ modulation, thus preventing neurological disorders

The miRNAs in the cortex and hippocampus of E18 fetuses that mother rats exposed to PM_2.5_ during pregnancy were assayed through a quantitative polymerase chain reaction for neurological disorders (autism spectrum disorders, schizophrenia, anxiety disorders, Tourette syndrome, Alzheimer disease, Prion diseases, Huntington disease, Parkinson disease, and Spinocerebellar ataxia type 1). Compared with the control group, the genes in the hippocampus and cortex of offspring whose mothers were exposed to PM_2.5_ were significantly modulated by air pollutants. These modulations could lead to neurological disorders such as schizophrenia, Prion diseases, and Alzheimer disease. The analysis results (Table 1) indicated that miRNAs in the offspring of pregnant rats had given GMI avoided modulation by PM_2.5_, making them less prone to neurological disorders.

**Table 1:** MiRNA analysis indicated that offsprings’ Schizophrenia, Prion disease and Alzheimer’s disease related RNA might be decreased; only rno-miR-9a-3p was not underwent this modulation.

Sample comparison		Potential disease
PM_2.5_ vs. Control	GMI + PM_2.5_ vs. PM_2.5_	
**Hippocampus**		
rno-miR-409a-3p↓		Schizophrenia
**Cortex**		
rno-miR-9a-3p↓	rno-miR-9a-3p↓	Schizophrenia
rno-miR-489-3p↓		Schizophrenia
rno-miR-30d-5p↓		Schizophrenia
rno-miR-346↓		Schizophrenia
rno-miR-339-5p↓		Prion Disease
rno-miR-191a-5p↓		Prion Disease
rno-miR-443-3p↓		Alzheimer
rno-miR-151-3p↓		Alzheimer
rno-miR-107-3p↓		Alzheimer
rno-miR-181a-5p↓		Alzheimer
rno-miR-139-5p↓		Alzheimer

## Discussion

PM_2.5_ is complex mixture whose core comprises organic carbon. Its particulates are less than 2.5 µm; it is the smallest substance that can be inhaled to cause severe damage. Once inhaled in the human body, PM_2.5_ increases the risk of chronic pulmonary diseases, cardiovascular diseases, stroke, and organ impairment^16,17^. The offspring of maternal mice exposed to PM_2.5_ from diesel exhaust tended to have more allergies^18^. This study revealed that after GMI administration, inflammation in mother rats throughout pregnancy eased, and WBC counts in their pups decreased (Figs 1 and 3). Moreover, according to our analysis of IgE and histamine levels in offspring GMI, especially at a dose of 0.33 μg/kg, reduced inflammatory response in rat offspring that was induced by their mothers’ exposure to PM_2.5,_ (Fig. 3). The expression of TNF-α, MCP-1, IL-1β, IL-15, and VEGF in rats increased significantly because of their exposure to PM_2.5_ during pregnancy but decreased after GMI administration (Fig. 2). Previous studies have indicated that exposure to PM_2.5_ after pregnancy causes an increase in the expression of proinflammatory factors (IL-1β, IL-6, and TNF-α)^19^, which in turn leads to substantial increases in the number of neutrophils^20^. MCP-1, produced by macrophages and endothelial cells, is a monocyte chemokine that facilitates the infiltration of macrophages into adipose tissues^21,22^. Accordingly, increased monocytes in white WBCs may be a result of increased MCP-1 expression. IL-15, produced by monocytes, facilitates the generation of natural killer cells and might result in increased lymphocyte counts after exposure to PM_2.5_. Increased VEGF expression causes vascular permeability^23^, as does exposure to PM_2.5_^24^; thus, PM_2.5_ passes through vessels to damage other tissues within the body. PM_2.5_ leads to increases in the expression of proinflammatory factors^19^, thus intensifying oxidative stress. This study revealed that GMI administration reduced the expression of proinflammatory factors^25^, inflammatory responses, and oxidative stress in rats, and attenuated their offspring’s inflammatory response.

The behavioral test results indicated that exposure to PM_2.5_ impaired long-term, working, and spatial memory in rat pups (Fig. 4). This finding corresponds with epidemiological findings that PM_2.5_ reduces working memory capacity^4,5^. After GMI administration, memory impairments due to PM_2.5_ in offspring improved. The hippocampus is responsible for cognition, learning, and memorization. The hippocampal circuitry receives information from CA3 and sends the information downwards. The circuit terminates at CA1. CA3 is responsible for spatial and working memory^26–29^. Long-term memory is the interaction between CA3 and CA1, which re-encodes and stabilizes information in the cortex^30–32^. The capability of neurons to process and transmit information depends on the complexity of dendritic branches. Dendritic branches in CA1 and CA3 decreased following exposure to PM_2.5_ (Fig. 5). The representative images of CA1 neurons show the dendritic branches number decrease in response to PM_2.5_ exposure, while GMI mitigates the branches number reduction (Fig. 5E–G). This finding agreed with the behavioral test results, in that the memory capacity of offspring declined because of exposure to PM_2.5_. Previous studies have suggested that exposure to PM_2.5_ increases oxidative stress in the amniotic fluid in the bodies of mothers, thus inhibiting the growth of the fetal brain^7^. By this reasoning, exposure to PM_2.5_ might have damaged the memory capacity of rat pups in this study through the same mechanism, and GMI administration might have prevented further PM_2.5_-induced increases in oxidative stress.

Microglia are immune cells in the central nervous system. As the results of the Western blot suggested (Fig. 6), high CD68 expression indicated an increased activation of microglia, and GMI administration attenuated this over activation. This increased microglial activation was probably due to elevated levels of inflammatory factors in peripheral tissues, which activate cerebral endothelial cells, affecting immune cells in the central nervous system^33^. Intense inflammation in offspring may arise from the inflammatory responses of mothers during pregnancy; moreover, the immature blood–brain barrier in the fetus leads to inflammation in its brain, thus inhibiting its development. Inflammation during pregnancy can lead to hippocampal abnormalities in the fetus, such as loss of neurons, astrocyte multiplication, and altered expression of neurotransmitter receptors^34^. Astrocyte multiplication causes microglia to increase, thus inducing neurological damage^35^; this accounts for neurological damage and memory impairment in rat offspring whose mothers are exposed to PM_2.5_ during pregnancy.

An miRNA expression analysis indicated that when mothers were administered GMI during pregnancy, miRNAs in offspring with schizophrenia, Prion diseases, and Alzheimer disease were not modulated by PM_2.5_; only rno-miR-9a-3p, which is related to schizophrenia, underwent this modulation (Table 1).

In summary, exposure to PM_2.5_ during pregnancy not only induces inflammatory responses in mother rats but also leads to memory impairment and cognitive decline in offspring as well as the overactivation of their microglia. GMI administration can alleviate the inflammatory responses, impaired memory capacity, and neurological damage induced by PM_2.5_. Therefore, despite exposure to PM_2.5_ during pregnancy causing long-term damage to the fetus, GMI administration can safeguard mothers and their offspring from PM_2.5_-induced damage.

## Supplementary information

Supplementary information
