# Supplementary material for: The Effect of Ganoderma Microsporum immunomodulatory proteins on alleviating PM2.5-induced inflammatory responses in pregnant rats and fine particulate matter-induced neurological damage in the offsprings
Source: Sci Rep. 2019 May 2;9:6854. doi: 10.1038/s41598-019-38810-5 (PMC6497649; doi:10.1038/s41598-019-38810-5)

The Effect of *Ganoderma Microsporum* immunomodulatory proteins on alleviating $PM_{2.5}$-induced inflammatory responses in pregnant rats and fine particulate matter-induced neurological damage in the offsprings

Chia-Yi Tseng[1,2,†], Jia-Yu Yu[3,†], Yu-Chen Chuang[1], Chia-Ying Lin[1], Chun-Hao Wu[1], Chia-Wei Liao[1], Fu-Hua Yang[1], Ming-Wei Chao[2,3]*

[1]Department of Biomedical Engineering, [2] Department of Bioscience Technology, [3]Center for Nanotechnology, Chung Yuan Christian University, Zhongli district, Taoyuan, Taiwan 320; Chung Yuan Christian University, Zhongli district, Taoyuan, Taiwan 32023

*Correspondence author to MWC at Department of Bioscience Technology, College of Science, Chung Yuan Christian University, 200 Chung Pei Road, Zhongli district, Taoyuan city, Taiwan 32023; Tel: 886-3-265-3512; Fax: 886-3-265-3599; Email: chao@cycu.edu.tw

[†]Authors contributed equally to the work.

Running title: GMI mitigates $PM_{2.5}$-induced inflammatory and neurological damages

Supplementary Figure 1. Raw data of western blot result for cortex and hippocampus.

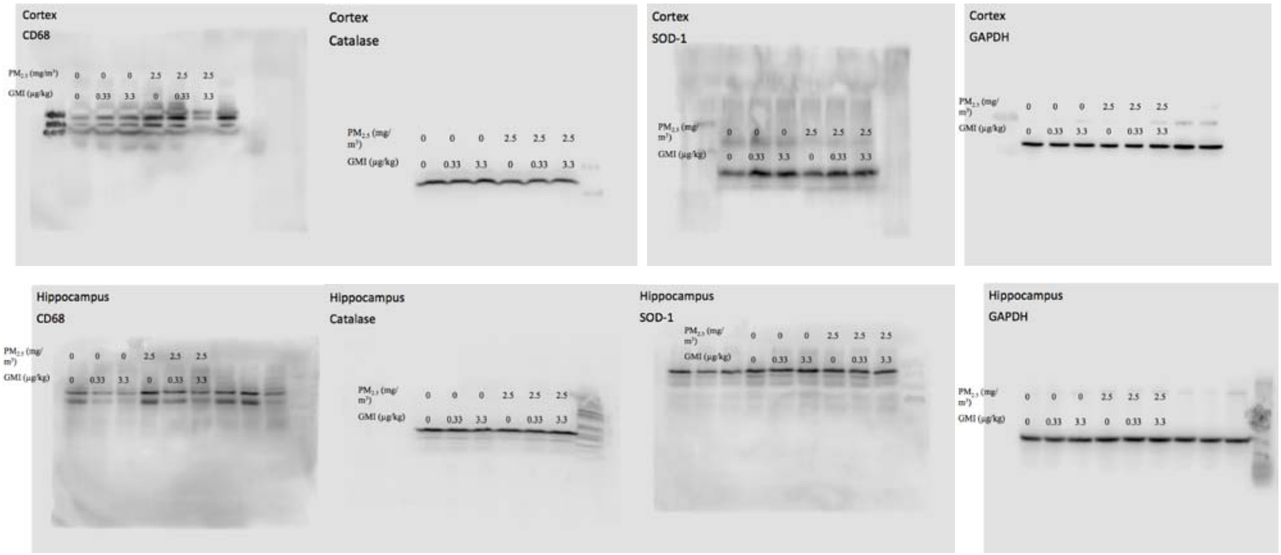

Supplement: Supplementary file 1 — Supplementary information [file 41598_2019_38810_MOESM1_ESM.pdf]
